# Templated‐Assembly of CsPbBr_3_ Perovskite Nanocrystals into 2D Photonic Supercrystals with Amplified Spontaneous Emission

**DOI:** 10.1002/anie.202006152

**Published:** 2020-08-13

**Authors:** David Vila‐Liarte, Maximilian W. Feil, Aurora Manzi, Juan Luis Garcia‐Pomar, He Huang, Markus Döblinger, Luis M Liz‐Marzán, Jochen Feldmann, Lakshminarayana Polavarapu, Agustín Mihi

**Affiliations:** ^1^ Institut de Ciència de Materials de Barcelona (ICMAB-CSIC) Campus de la UAB 08193 Bellaterra Catalonia Spain; ^2^ Chair for Photonics and Optoelectronics Nano-Institute Munich Department of Physics Ludwig-Maximilians-Universität (LMU) Königinstrasse 10 80539 Munich Germany; ^3^ Department of Chemistry Ludwig-Maximilians-Universität (LMU) Butenandtstrasse 5–13 (E) 81377 Munich Germany; ^4^ CIC biomaGUNE Basque Research and Technology Alliance (BRTA) Paseo de Miramón 182 20014 Donostia—San Sebastián Spain; ^5^ Centro de Investigación en Red en Bioingeniería Biomateriales y Nanomedicina (Ciber-BBN) Paseo de Miramón 182 20014 Donostia—San Sebastián Spain; ^6^ Ikerbasque, Basque Foundation for Science 48013 Bilbao Spain

**Keywords:** 2D photonic crystals, amplified spontaneous emission (ASE), PDMS template, perovskite nanocrystals, self-assembly

## Abstract

Perovskite nanocrystals (NCs) have revolutionized optoelectronic devices because of their versatile optical properties. However, controlling and extending these functionalities often requires a light‐management strategy involving additional processing steps. Herein, we introduce a simple approach to shape perovskite nanocrystals (NC) into photonic architectures that provide light management by directly shaping the active material. Pre‐patterned polydimethylsiloxane (PDMS) templates are used for the template‐induced self‐assembly of 10 nm CsPbBr_3_ perovskite NC colloids into large area (1 cm^2^) 2D photonic crystals with tunable lattice spacing, ranging from 400 nm up to several microns. The photonic crystal arrangement facilitates efficient light coupling to the nanocrystal layer, thereby increasing the electric field intensity within the perovskite film. As a result, CsPbBr_3_ 2D photonic crystals show amplified spontaneous emission (ASE) under lower optical excitation fluences in the near‐IR, in contrast to equivalent flat NC films prepared using the same colloidal ink. This improvement is attributed to the enhanced multi‐photon absorption caused by light trapping in the photonic crystal.

## Graphical abstract

**Perovskite nanocrystals** (NCs) are self‐assembled into 2D photonic supercrystals using pre‐patterned polydimethylsiloxane (PDMS) templates and their optical spectra are tunable by varying the lattice spacing. These photonic crystals show amplified spontaneous emission (ASE) under lower optical excitation fluences in the near‐IR region due to enhanced multi‐photon absorption caused by light trapping in the photonic crystal.

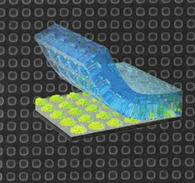

## Introduction

Halide perovskite nanocrystals (NCs)^1^ have emerged as a new class of efficient and tunable light sources for various optical and photonic applications and have already shown great promise in solar cells, photodetectors, LEDs and lasers.1b, 2 The optical properties of halide perovskite NCs are readily tunable across the entire visible light spectrum, by means of their halide composition as well as their dimensionality.1c, 3 We have witnessed tremendous research progress regarding the shape and composition‐controlled synthesis of perovskite NCs for tailoring their emission color, as well as improving quantum efficiency.3f, 4 Among all available compositions, Pb‐based perovskite NCs stand out for their stability and optical efficiency, even as a two‐photon‐pumped lasing medium.^5^ Because of the interest in perovskite nanocrystals as light sources, a light management strategy is often required. An appealing strategy to engineer the optical response of the material without recurring to changes in its composition comprises molding it into higher order architectures. This approach has been previously applied to colloidal gold nanocrystals, self‐assembled into 2D plasmonic supercrystals (SCs) exhibiting tunable lattice plasmon resonances, but also to PbTe nanocrystals, for which the arrangement was modulated into a variety of motifs.^6^ Self‐assembly concepts have also been recently extended to colloidal perovskite NCs,^7^ including the formation of colloidal SCs with distinct optical properties.4b, 8 Despite these early efforts, the self‐assembly of perovskite NCs into 2D SC arrays with subwavelength patterns remains a daunting challenge, and therefore their optical properties remain unexplored.

Photonic crystals (PhCs) are materials exhibiting a periodicity in the refractive index on the order of the wavelength of incident light.^9^ Light propagation in these materials can be described in terms of photonic bands and band gaps (forbidden intervals). PhCs, however, also exhibit many other interesting phenomena. 2D photonic crystals, very much as other gratings, can diffract light very efficiently, which has been used e.g., to enhance the optical path in solar cells.^10^ Many materials can be organized into photonic crystals by periodically arranging them in the sub‐micrometer range and thereby exhibit optical properties differing from those of the bulk material.6b, 11 In this work, we propose a simple and scalable method to fabricate large area 2D photonic crystals using colloidal solutions of perovskite nanocubes. We used template‐induced self‐assembly to produce large‐area arrays from colloidal perovskite NC dispersions. The obtained perovskite 2D PhCs have lattice parameters on the order of visible light wavelengths, with a strong iridescence indicating the high quality of the structure. Furthermore, the photonic architecture is engineered to couple the 800 nm pumping light into photonic modes propagating in the perovskite PhC layer. This enhanced electric field intensity in the material, as revealed by FDTD simulations, is used in turn to produce amplified spontaneous emission (ASE) in PhC films under fluence that is too low to obtain ASE from planar films obtained under similar conditions. The combination of the NCs optical properties with the photonic environment provides an efficient use of optical pumping to boost the light intensity‐dependent mechanism of two‐photon absorption.

## Results and Discussion

**Figure 1:**
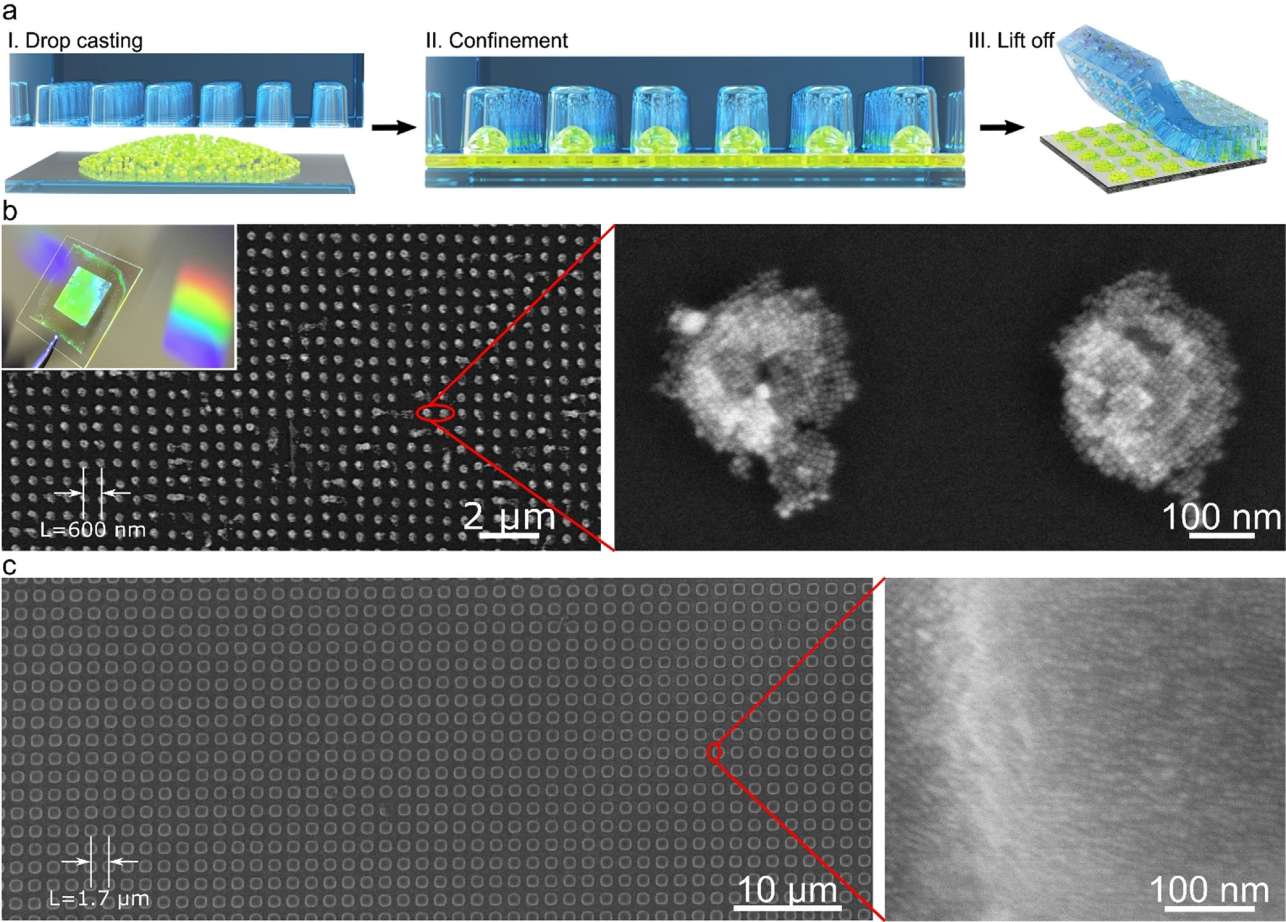
a) Schematic illustration of PDMS template‐assisted fabrication of 2D photonic supercrystals made of perovskite NCs. b),c) Representative SEM images of 2D photonic supercrystals made of CsPbBr_3_ arrays with lattice parameters of 600 nm and 1700 nm, respectively. The inset in (b) is a photograph of different CsPbBr_3_ SC arrays on glass substrates, displaying diffraction under white light illumination.

The colloidal CsPbBr_3_ nanocube ink used for the fabrication of 2D photonic crystals was prepared by ligand‐assisted ultrasonication (see experimental section in the Supporting Information SI). The synthesized NCs are highly monodisperse, with an average size of 10.7±0.9 nm (Figure S1). The colloidal dispersion was purified by centrifugation to obtain a perovskite NC ink in hexane at a 100 mg mL^−1^ concentration. The perovskite NCs exhibited green photoluminescence (PL) with a maximum at 513 nm (Figure S1). Figure 1 a illustrates the self‐assembly process using poly(dimethylsiloxane) (PDMS) templates^12^ with pre‐patterned square arrays (see AFM image of the PDMS surface in Figure S2).

The modified template‐assisted self‐assembly process is illustrated in Figure 1. In the first step, a drop (10 μL) of CsPbBr_3_ colloidal dispersion is deposited on a glass substrate and immediately covered with a PDMS template with holes facing the droplet (Figure 1 a‐I). The resulting confinement forces the colloidal dispersion into the holes of the PDMS template, from where the solvent is left to evaporate for 1 hour (Figure 1 a‐II). Homogeneous distribution of the dispersion and conformal contact between the substrate and the PDMS mold was ensured by placing a 700 g weight on top of the stamp during the entire drying step (see experimental section in SI). Finally, the PDMS mold is gently lifted‐off from the substrate, yielding an array of NCs with the negative image of the template pattern on the substrate (Figure 1 a‐III). The obtained PhC films exhibit strong green PL under UV light illumination (see left inset in Figure 4 b below) and appear iridescent under white light illumination as shown in the inset of Figure 1 b. The diffraction of white light into different colors originates from the subwavelength periodicity of the NC assemblies on the substrate. The 2D perovskite photonic crystals were further characterized by scanning electron microscopy (SEM). SEM images of the films fabricated using a PDMS template with a lattice parameter (L) of 600 nm clearly show large‐area 2D arrays of NC assemblies (Figure 1 b). Interestingly, the CsPbBr_3_ NCs in the assemblies exhibit cubic close‐packing into an ordered supercrystal, as revealed by high magnification SEM images (Figure 1 b). The lateral dimension of the supercrystal is ≈200 nm. These supercrystals are formed during evaporation of the solvent inside the holes of the PDMS template. However, parameters such as ligand concentration and solvent composition should be investigated further to better understand the mechanism of supercrystal formation under the template assembly.6b

Perovskite photonic crystals with different lattice parameters were fabricated by different choice of PDMS molds. Optimization of the system allowed us to successfully assemble the NCs into structures with L ranging from 1700 nm down to 400 nm, thereby overcoming the increasing difficulty involved in pattern miniaturization. SEM images of PhCs with different lattice parameters are shown in Figure S3. Bright field optical microscopy overview images of the samples confirm the formation of uniform and large‐area 2D PhCs (Figure S4). Shown in Figure 1 c is a representative SEM image of a perovskite 2D PhC with a lattice parameter of 1700 nm, prepared on a glass substrate using the corresponding PDMS template. In this particular array, high magnification SEM reveals that the NCs are closely packed within individual SCs (Figure 1 c). In order to better visualize the individual CsPbBr_3_ NCs, we assembled the same NCs over carbon‐coated grids and analyzed them by transmission electron microscopy (TEM, Figure S5). TEM images of these assemblies and their 2D fast Fourier transform (FFT) analysis indeed confirm the formation of self‐assembled perovskite supercrystal arrays (Figure S5). In addition, the assemblies can be seen on the substrate as well after a thorough washing of the surface with methylacetate or acetone (Figure S6). However, this process partially destroys the NCs and the assemblies.

**Figure 2:**
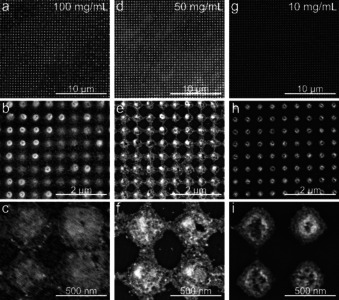
SEM characterization of photonic crystals prepared by self‐assembly of NCs from dispersions at concentrations of a)–c) 100 d)–f) 50 and g)–i) 10 mg mL^−1^. The concentration of NCs and ligands has a strong influence on the viscosity of the sample, altering the distribution of NCs along the surface. Isolated pillars are formed at low concentrations, while a residual layer of NCs is formed all over the sample at 100 mg mL^−1^.

**Figure 3:**
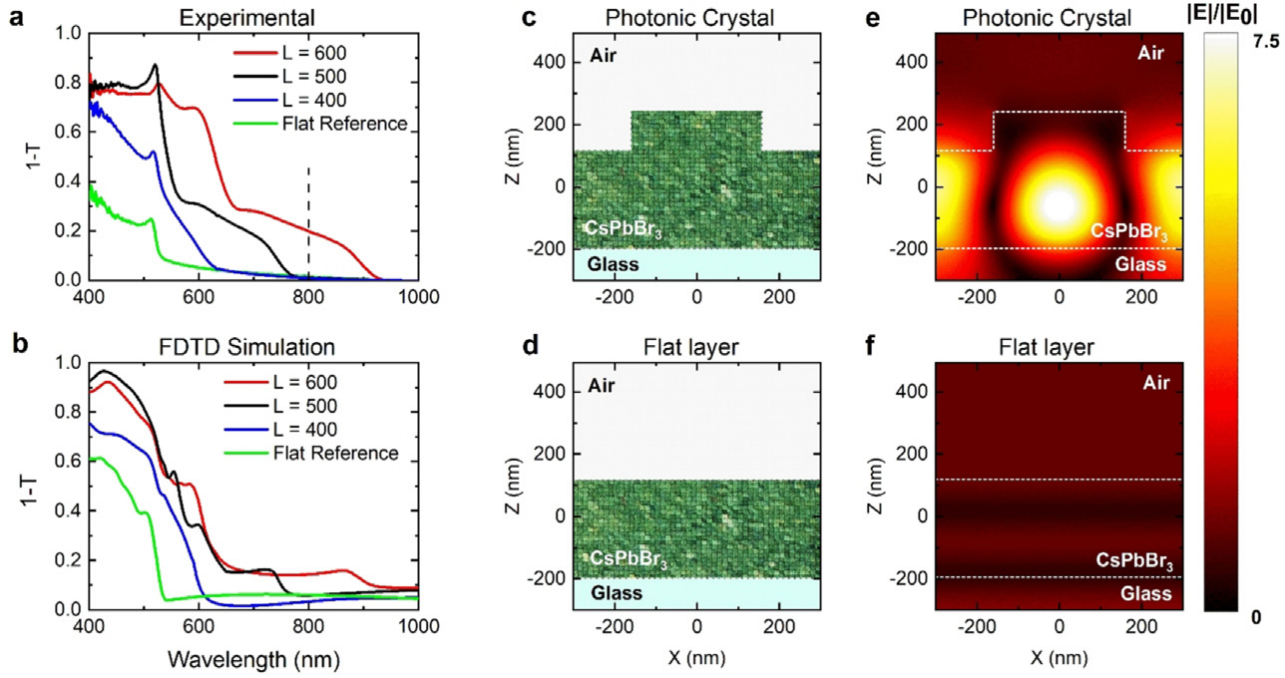
Lattice resonances and field enhancement: Experimental (a) and theoretical (b) extinction (1−*T*, where *T* is transmittance) spectra for a NC thin film (green curve) and for different PhCs with lattice parameters of 400 (blue), 500 (black) and 600 nm (red). The vertical dashed line in (a) corresponds to the 800 nm laser excitation wavelength used in the nonlinear experiments. c)–f) FDTD simulated spatial distribution of the electric field enhancement illustrating the effect of light diffraction, for the case of 600 nm lattice parameter versus a perovskite flat film (e,f).

In addition to the dimensions of the template, we found that both concentration and viscosity of the CsPbBr_3_ colloidal dispersion play a critical role on the final structure of the PhC. We fabricated a series of photonic crystals with fixed geometrical parameters and varying concentration of NCs (Figure 2). We observed that isolated SC pillars were effectively achieved with concentrations below 10 mg mL^−1^, whereas increasing the concentration of nanocrystals resulted in residual nanocubes connecting the pillars. For the highest NC concentrations (80–100 mg mL^−1^) the final photonic architecture was composed of an array of CsPbBr_3_ nanopillars on top of a residual layer of nanocubes. The height of the pillars is in the range of 80–100 nm (Figure S6). This latter configuration is particularly useful in the case of light emission studies (amplified spontaneous emission and lasing), where thick layers of active material are required. We focus now on the optical properties of perovskite 2D photonic crystals with lattice parameters of 400, 500 and 600 nm, produced from 100 mg mL^−1^ dispersions. These PhCs consist of arrays of 100 nm high pillars, on top of a 250 nm flat film of NCs, as schematically illustrated in Figure 3 c. This film thickness is required to observe a meaningful photoluminescence signal from the sample. We first inspect the extinction (1—transmittance) from the PhCs and compare it to the extinction from a flat NC film (Figure 3 a).

The flat reference sample shows an excitonic peak at 512 nm, while PhCs with lattice parameters of 400, 500 and 600 nm exhibit excitonic peaks at 515, 519 and 527 nm, respectively. However, a series of new peaks appear for the patterned samples, which redshift with increasing lattice spacing. These features are Rayleigh anomalies associated to the offset of diffraction. These peaks indicate that light impinging normally to the sample is diffracted by the periodicity of the array.6b We carried out finite difference time domain (FDTD) simulations from Lumerical Solutions Inc., to theoretically obtain the corresponding extinction curves (Figure 3 b) and to extract the spatial distribution of 800 nm incident light on a 600 nm lattice parameter perovskite PhC, versus a flat film (see numerical details in SI). The field profiles reveal that the electric field within the photonic crystal is 7.5 times higher than that in a flat film of the same thickness (Figure 3 c–f). Effectively, a perovskite PhC diffracts the incoming light and couples it to the perovskite residual layer under the pillars. This light trapping strategy is typically used in photovoltaics to couple light more efficiently to the active layer of a solar cell, by increasing the optical path of light in the material.^10^ Similarly, this scheme has been exploited in the literature to enhance the two‐photon absorption (TPA) of light emitters, a third order process whose absorption cross section scales non‐linearly with the intensity of the incoming light (see numerical details in SI).^13^ However, unlike previous reports in which quantum dot‐decorated PhCs^14^ were fabricated by costly lithographic techniques such as electron beam lithography (EBL), our approach readily produces a high quality PhC configuration within minutes. We thus applied this strategy to optimize the use of the 800 nm pumping light and look for an improved PL enhancement, following recent studies on bulk‐perovskite metasurfaces that showed enhanced linear and nonlinear PL.2a, 11

**Figure 4:**
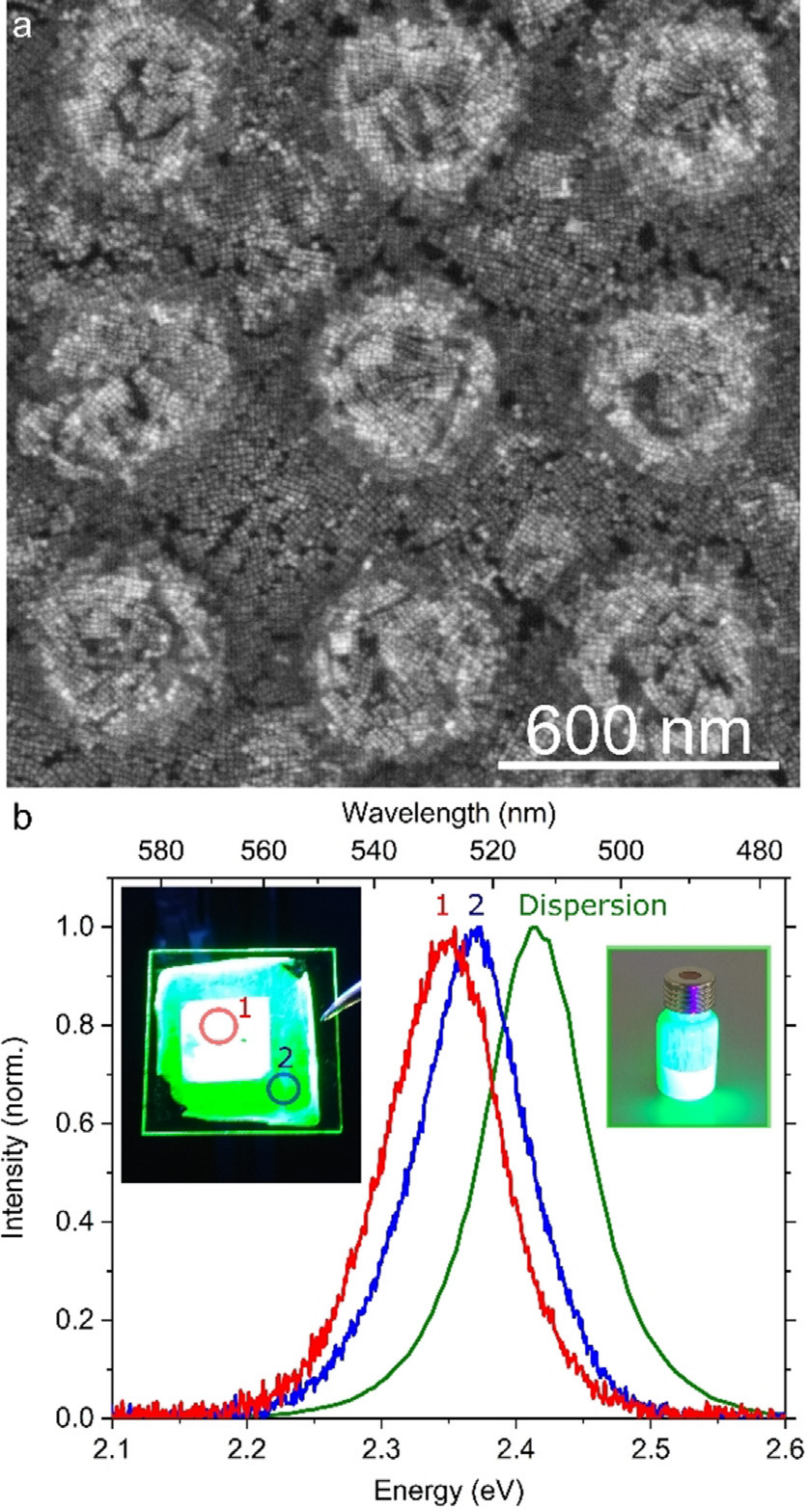
a) SEM close view of SC arrangement in a perovskite PhC obtained from a 100 mg mL^−1^ NC dispersion, used for ASE studies. b) Photoluminescence spectra obtained from inside the patterned area (red), outside of the patterned area (blue) and from the initial colloidal dispersion (green).

We selected the perovskite PhCs fabricated from 100 mg mL^−1^ dispersions (Figure 4 a) for PL characterization, since these resulted in the thicker architectures reproducing the light trapping scheme shown in Figure 3 c. We collected PL under 400 nm excitation from the PhC, as well as from a flat region outside of the patterned area, and compared it with the PL from the NC dispersion (Figure 4 b). The NCs in dispersion have a PL centered at 513 nm, with a Gaussian FWHM of 21 nm. Instead, both the NCs in the PhC pattern and in the flat reference region exhibited a spectrally redshifted emission with respect to the PL of the NC dispersion, respectively at 528 nm (FWHM: 23 nm) and 524 nm (FWHM: 23 nm). Importantly, this redshift is not only observed in the emission but also in the extinction spectra (Figure 3 a), as a redshift of the 1s excitonic transition. Such a redshift is known to stem from self‐assembly of NCs into colloidal superlattices.^8^, ^15^ The physical origin of the redshifted PL stems from the close vicinity of neighboring NCs, which leads to overlap of their electronic wavefunctions. This coupling phenomenon between NCs leads to the formation of minibands, both in the conduction and in the valence bands. The split energy levels result in a lower energy for excitonic absorption and recombination (i.e., the observed redshifts).8a In our case, the redshifted absorption and emission from the PhCs underlines a higher degree of electronic coupling in such structures, with respect to the NCs in the reference flat area. Therefore, the SCs obtained inside the holes of the PDMS templates are of higher quality than those spontaneously formed in the unpatterned area, which evidences the potential versatility of this fabrication technique.

**Figure 5:**
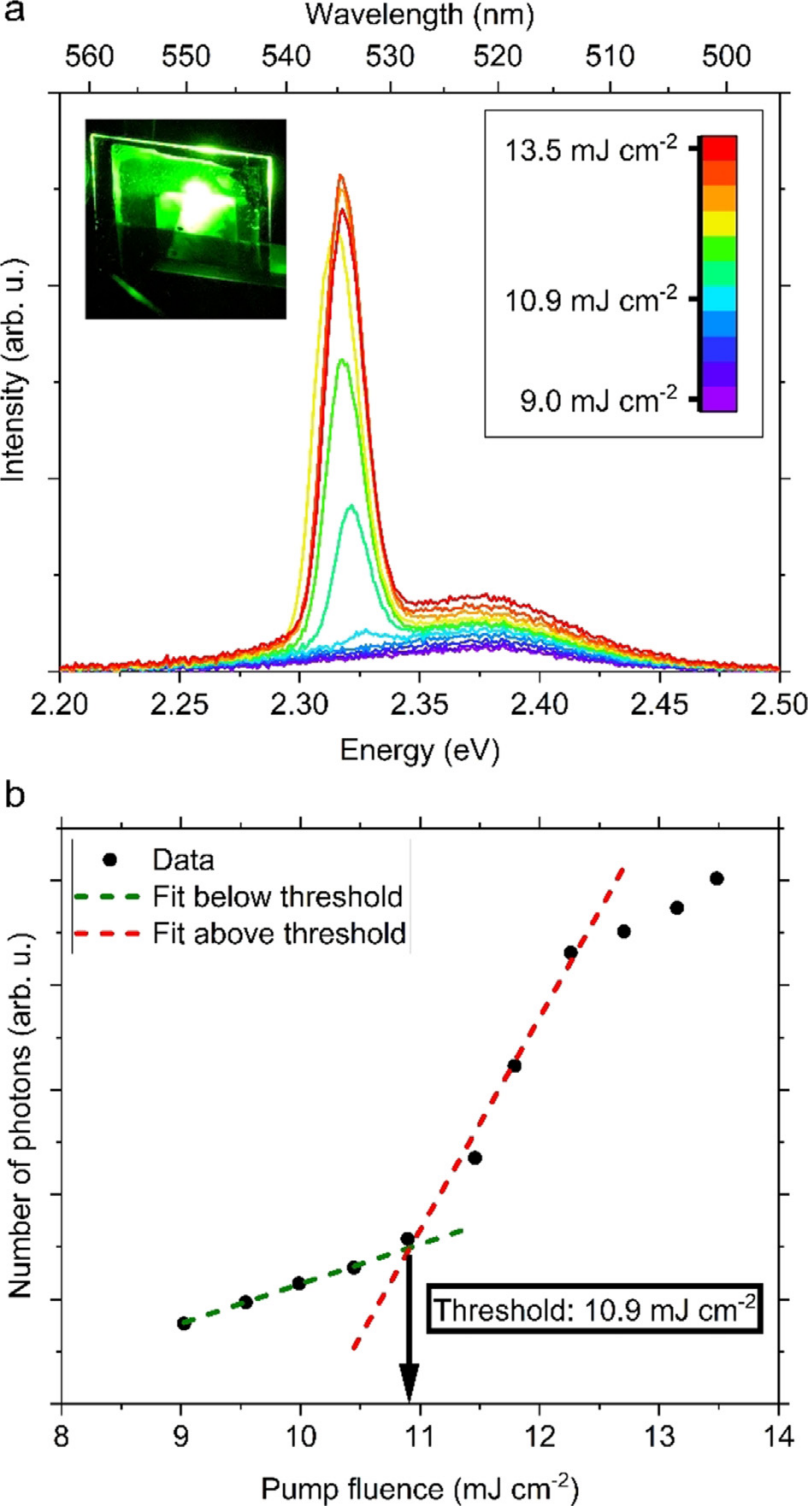
a) Fluence‐dependent PL (evolution of ASE) of CsPbBr_3_ 2D photonic crystals film and b) the corresponding emission intensity obtained by integration of the spectra. The inset in (a) is a photograph of the photonic crystal film showing waveguiding above excitation threshold for ASE. The dotted lines in (b) are the best linear fits for intensities below (green) and above (red) threshold. The spectra were acquired with 800 nm femtosecond laser excitation.

Finally, to probe the effect of the field enhancement obtained by the presence of PhCs on the light emitting properties of perovskite NCs, we photoexcited the system with an 800 nm pulsed laser system, with a repetition rate of 1 kHz and a pulse width of 100 fs. This excitation wavelength corresponds to the electric field spatial distribution observed for the PhCs and is attributed to the increase in light diffraction, which is absent for unpatterned NCs (see Figure 3 a). CsPbBr_3_ NC films have been shown to exhibit below band gap absorption and nonlinear amplified spontaneous emission (ASE).^16^ We therefore explored the possibility to obtain ASE through nonlinear photon absorption in our PhC pattern, varying the intensity of the photoexcitation and comparing the observation to the ASE obtained from unpatterned NCs. ASE was reproducibly observed from our PhC pattern (see Figure 5 a), as evidenced by the emergence of an additional narrow emission peak on the low‐energy side of the PL spectra. By integrating the PL intensity versus the pump fluence we obtained an ASE threshold intensity of 10.9 mJ cm^−2^ (Figure 5 b). Employing an unpatterned film from the same NC ink as a reference, we observed no ASE, even for the maximum excitation fluence of 13 mJ cm^−2^ (Figure S7). These results suggest that 2D perovskite PhCs make more efficient use of the excitation light by coupling to photonic modes with more intense field profiles at 800 nm, as predicted from FDTD simulations (Figure 3 e). This results in a decrease of the photoexcitation intensity necessary to achieve population inversion, hence ASE. This finding indicates how PhCs can be employed to tailor the field enhancement effects in optoelectronic devices based on perovskite NCs. We envision that the optimized use of the optical pumping provided by the ordered nanostructuring of the NC ink not only broadens the range of applicability of this material beyond its visible band gap, but also will be particularly beneficial to light emitting applications under NIR excitation.

## Conclusion

In conclusion, we have demonstrated the fabrication of large‐area CsPbBr_3_ 2D photonic crystals by template‐assisted self‐assembly, using 10 nm nanocrystal inks. We produced homogeneous photonic architectures with lattice parameters ranging from 400 nm to 1700 nm. The PhCs obtained exhibit optical resonances in the extinction spectra under normal incidence produced by the onset of diffraction from the photonic crystal geometry. These resonances can be tuned through the choice of lattice parameter (L) in the architecture. We reproduced the experimental spectra using FDTD simulations and investigated the field distribution in the structure, revealing a strong field enhancement at below band‐gap energies. Furthermore, we have taken advantage of this light trapping scheme to enhance the two‐photon absorption of NIR light and obtained ASE. We anticipate that these results will have broader impact in the field of perovskites beyond ASE and open new opportunities to tailor the optical properties of this novel material at the nanoscale.

## Conflict of interest

The authors declare no conflict of interest.

## Supporting information

As a service to our authors and readers, this journal provides supporting information supplied by the authors. Such materials are peer reviewed and may be re‐organized for online delivery, but are not copy‐edited or typeset. Technical support issues arising from supporting information (other than missing files) should be addressed to the authors.

Supplementary
