# Supplementary material for: Templated‐Assembly of CsPbBr3 Perovskite Nanocrystals into 2D Photonic Supercrystals with Amplified Spontaneous Emission
Source: Angew Chem Int Ed Engl. 2020 Aug 13;59(40):17750–6. doi: 10.1002/anie.202006152 (PMC7540499; doi:10.1002/anie.202006152)
Supplement: Supplementary file 1 — Supplementary [file ANIE-59-17750-s001.pdf]

Angewandte Chemie
Eine Zeitschrift der Gesellschaft Deutscher Chemiker

Supporting Information

Templated-Assembly of $CsPbBr_3$ Perovskite Nanocrystals into 2D Photonic Supercrystals with Amplified Spontaneous Emission

David Vila-Liarte[+], Maximilian W. Feil[+], Aurora Manzi, Juan Luis Garcia-Pomar, He Huang, Markus Döblinger, Luis M Liz-Marzán, Jochen Feldmann, Lakshminarayana Polavarapu, and Agustín Mihi**

anie_202006152_sm_miscellaneous_information.pdf

SUPPORTING INFORMATION

Table of contents

1. Experimental Section 2

2. Suppression of the Coffee-Ring effect..7

3. Design of the Optical Response of the Photonic Crystals 8

4. $CsPbBr_3$ Nanocrystal Characterization 10

5. PDMS mold 11

6. Low magnification images of Perovskite Photonic Crystals.................................12

7. TEM and SEM inspection of Perovskite Photonic Crystals.................................13

8. Photoluminescence of a flat NC film under 800 nm excitation 15

9. References 16

1. Experimental Section

Materials: For the synthesis of $CsPbBr_3$ nanocrystals: Cs_2CO_3 (Sigma Aldrich, 99.9% trace metals basis), $PbBr_2$ (Sigma Aldrich, 99.999% trace metals basis), oleylamine (Sigma Aldrich, ≥ 98% primary amine), oleic acid (Sigma Aldrich, technical grade 90%), hexane (Sigma Aldrich, anhydrous 95%). For the polydimethylsiloxane (PDMS) preparation: trichloro(1H,1H,2H,2H-perfluorooctyl) silane (PFOTS) (Alfa Aesar, 97%), soft PDMS Sylgard 184 kit (Dow Corning), hard-PDMS (h-PDMS) kit (Gelest). Acetone, isopropanol and ethanol (Labkem). All chemicals were used as received.

$CsPbBr_3$ nanocrystals synthesis and purification[1]: The synthesis is performed by weighting 1 mmol Cs_2CO_3 and 3 mmol of $PbBr_2$ powders and put it into a 20 mL glass bottle. Afterwards, 10 mL 1-octadecene, followed by 1.5 mL oleic acid and 1.5 mL oleylamine were added. The obtained reaction mixture was immediately put into a tip sonicator (SONOPULS HD 3100, BANDELIN) using a power of 30 W kept for 30 min. Eventually, the obtained deep yellow reaction mixture was taken out of the tip sonicator and cooled down to room temperature by ambient atmosphere. Finally, the dispersion was centrifuged (Eppendorf Centrifuge 5810 R) at a speed of 6000 rpm for 15 min. The supernatant was discarded and the thus obtained precipitate was redispersed in 3 mL of hexane to obtain a very dense nanocube dispersion. For the purification following the synthesis, 25 ml hexane, 0.1 mL oleic acid and 0.1 mL oleylamine were added to 0.25 mL of the nanocube dispersion. This way, large aggregates of nanocubes formed during the synthesis are disassembled partly into separated nanocubes. This diluted dispersion is afterwards centrifuged two times and each time the precipitate is discarded. The first centrifugation is performed with a speed of 1000 rpm for 10 min and the second centrifugation with 12000 rpm for 10 min. Afterwards, 15 mL of the resulting dispersion was equally split into 10 centrifuge tubes and centrifuged (Eppendorf miniSpin plus) with

14000 rpm for 60 min. The supernatant was discarded for all tubes and the precipitate was dispersed in 0.1 mL hexane. The thus obtained 10 samples were merged to a 1 mL final sample which was used for the assembly of photonic crystals.

***PDMS Mold Preparation**:* Patterned molds of PDMS were obtained by the replication of silicon masters engraved with the original pattern. Composite PDMS molds were utilized in this work to obtain high fidelity replicas.[2] The silicon masters were functionalized with PFOTS (see materials) by vapor phase silanization using 10 μl PFOTS in a sealed vessel. Upon complete evaporation after 30 minutes under vacuum, the excess of PFOTS on the silicon surface was removed by rinsing the master with abundant isopropanol and heating up to 130 ºC for 20 minutes. Briefly, a thin h-PDMS film was firstly used to replicate the nanostructure followed by drying for an hour and 60 °C curing for another hour. Then, a thick supporting soft-PDMS layer of around 5 mm of thickness is added to the h-PDMS to allow handling and to confer flexibility. Soft-PDMS was used by pouring a 10:1 mixture of prepolymer and curing agent. The mixture was previously mixed and degassed under vacuum in a desiccator to remove the trapped air to achieve homogeneous density. Finally, soft-PDMS was cured at 80 °C for 2 hours and the mold is gently detached from the master. The templates obtained by this procedure present the same dimensions of the original silicon masters.[3] The details of the available nanostructures are listed below.

Table 1: Dimensions of the different patterns: lattice parameter of the square array of nanoholes, diameter and depth of the cavities, and total patterned area of the template.

Lattice parameter (nm)	Diameter (nm)	Depth (nm)	Patterned area (cm^2)
**400**	300	350	1
**500**	300	350	1

**600**	300	350	1
**1000**	600	250	0.5
**1600**	960	250	0.5
**1700**	1020	250	0.5

Substrate Preparation: Glass coverslips of 22 x 22 mm^2 (Menzel, #15) are used as target substrates. Standard cleaning protocol consisted in rubbing with a 2% Hellmanex III solution and rinsing with excessive deionized water, acetone, isopropanol and then dried with compressed air. Prior to assembly, clean substrates are fixed onto a leveled table inside the fume hood with adhesive tape.

Template-Assisted Self-Assembly of $CsPbBr_3$ Nanocrystals: A droplet of 5-10 μL of nanocrystals dispersion is drop casted at the center of the substrate and quickly covered by a PDMS mold. Immediately after that, a weight of 700 g is carefully placed on top of the PDMS to apply extra pressure. Upon complete evaporation, weight is lifted off without disturbing the position of the mold. Then, PDMS template is gently detached from the fixed substrate at lowest rate possible to minimize disruption of the assembled structures.

PDMS templates can be reused several times by simply removing attached particles with adhesive tape and ultrasonication in ethanol. It is advisable to observe the mold under UV-light to identify the presence of perovskite nanocrystals on it. Importantly, infiltration of organic solvents into PDMS may produce mechanical deformation of the pattern due to swelling of the polymeric structure. In our case deformation of the PDMS molds was only observed in templates with very low fill fraction of PDMS; that is with features in which the walls of PDMS were below 100 nm thick (such as 400 nm lattice parameter

and 300 nm hole diameter). Nevertheless, the quality of the prepared samples is not affected (see section S5), and the mold recovers its original shape after complete drying of the organic solvent (see section S3).

Characterization: Scanning Electron Microscopy (SEM) images of the insulating samples were successfully acquired without prior sputtering using a FEI Helios operated with an acceleration voltage of 2 kV. Zeiss Gemini Ultra Plus SEM was also employed at acceleration voltage of 3 kV. Transmission Electron Microscopy characterization was performed with a bright field JEOL JEM-1011 with 80-100 kV of accelerating voltage.

Fourier Transformed Infrared (FTIR) characterization was performed with a FTIR spectrometer (Vertex 70 Bruker) attached to an optical microscope (Hyperion, Bruker) for far-field transmission measurements in the 400 to 1200 range. All the spectra were collected with a 4x objective.

Absorbance of colloidal dispersions were taken with a Cary 5000 UV-Vis-NIR spectrophotometer.

For Photoluminescence (Figure 4) and amplified spontaneous emission (Figure 5) measurements an 800 nm femtosecond pulsed Coherent Inc. laser system (pulse width: 100 fs, repetition rate: 1 kHz) was employed. The photoluminescence at low pump fluence was measured with 400 nm excitation laser light using second harmonic generation of a barium borate crystal. Photoluminescence and amplified spontaneous emission were collected with a Thorlabs CCS200 CCD spectrometer. The photoluminescence of the nanocrystal dispersion was measured using a Cary Eclipse Fluorescence Spectrophotometer from Agilent Technologies.

Atomic Force Microscopy profile of the patterned surface of the PDMS mold was obtained with an Agilent 5100.

2. Suppression of the Coffee-Ring effect

The main challenge regarding pattern formation upon evaporation[4] is to overcome the formation of the so-called coffee-ring effect (CRE),[5] which is originated from the outwards flows that arise to replenish the evaporated solvent at the pinned liquid-air contact line. Such flows of solvent can carry most of the particles to the rim of the wetted area giving rise to an inhomogeneous distribution of material and producing uneven surfaces.

In this work, the self-assembly process has been optimized so no modifications of the surface chemistry are needed to overcome the coffee-ring effect. The inhomogeneous distribution of NCs that CRE could cause was counterbalanced by two facts: i) first by applying pressure on top of the PDMS mold immediately after drop casting. This way, the dispersion was homogeneously spread along the substrate since the beginning of the process. And ii) the infiltration of hexane into PDMS[6] provides an alternative drying route and therefore further preventing the formation of CRE during evaporation, keeping the initial distribution constant.

3. Design of the Optical Response of the Photonic Crystals

Finite Differences Time Domain (FDTD) simulations: Numerical calculations were performed using Lumerical FDTD solutions (www.lumerical.com). A polarized plane wave source impinging at normal incidence to different NC clusters was modeled in a square unit cell with periodic boundaries. The refractive index and the dispersive extinction coefficient of $CsPbBr_3$ are obtained from literature.[7] The glass substrate is set with a refractive index of n_{glass} = 1.43.

For the Figure 3b of the manuscript, only the normal diffraction order has been considered to achieve similar conditions to the experimental set up. Consequently, different plateaus in extinction appear in higher wavelengths where the absorption of the sample is null. The position of the steps for the different photonic crystals with lattice parameter L follow the equation:

$$\lambda \sim L n_{glass} \left(\sqrt{p^2 + q^2}\right)^{-1}$$

where p and q are integers: $p, q = 0{,}1, 2 \ldots$

In order to maximize the 2-photon absorption of an 800 nm light excitation, we needed to engineer a photonic crystal coupling the waveguide modes in the perovskite residual layer with the diffraction grating equation. An approximation for the wavelengths of the coupled resonances, in the limit of linear and shallow grating depth, is possible using the equations of Ref. 8,[8] which depend on the thickness of the residual layer t, the lattice parameter L and the respective refractive index of the superstrate, the perovskite layer, and substrate.

For L=*600 nm* and a thickness of the residual layer of t= *300 nm* the diffraction grating is coupled with an electric field waveguide order for *λ~800 nm.*

Relationship between absorption and intensity of the incident light: The absorption per unit volume for monochromatic waves with frequency, ω can be calculated from:

$$P_{abs} = -\frac{1}{2} Re(i\omega \boldsymbol{E} \cdot \boldsymbol{D}) \quad (1)$$

where $\boldsymbol{E}$ and $\boldsymbol{D}$ are the electric field and the electric displacement, respectively.

If we consider only dissipative processes in the expression for $\boldsymbol{D}$,

$$\boldsymbol{D} = \varepsilon_0 (\varepsilon_r + \chi^{(3)} |\boldsymbol{E}|^2) \boldsymbol{E} \quad (2)$$

the first term involving describes a linear absorption. The even-order susceptibilities like, $\chi^{(2)}$, $\chi^{(4)}$ etc, do not make a contribution to the dissipative processes in our case. Therefore, the lowest-order non-linear absorption will be described by the imaginary part of $\chi^{(3)}$, which corresponds to two-photon absorption. From Eqs. (1) and (2) if we define I as the intensity of light and is defined as $I = |\boldsymbol{E}|^2$, it should be noted that the energy absorption in this non-linear absorption process is not linearly but quadratically dependent on the light intensity.

4. **CsPbBr3 nanocrystals characterization**

The NCs obtained by ligand-assisted ultrasonication are highly monodisperse with an average size of 10.7 nm. The colloidal dispersion was purified by centrifugation to obtain perovskite NC ink redispersed in hexane at a 100 mg/mL concentration. The perovskite NCs exhibited green photoluminescence (PL) with a maximum at 513 nm with a Gaussian FWHM of 21 nm.

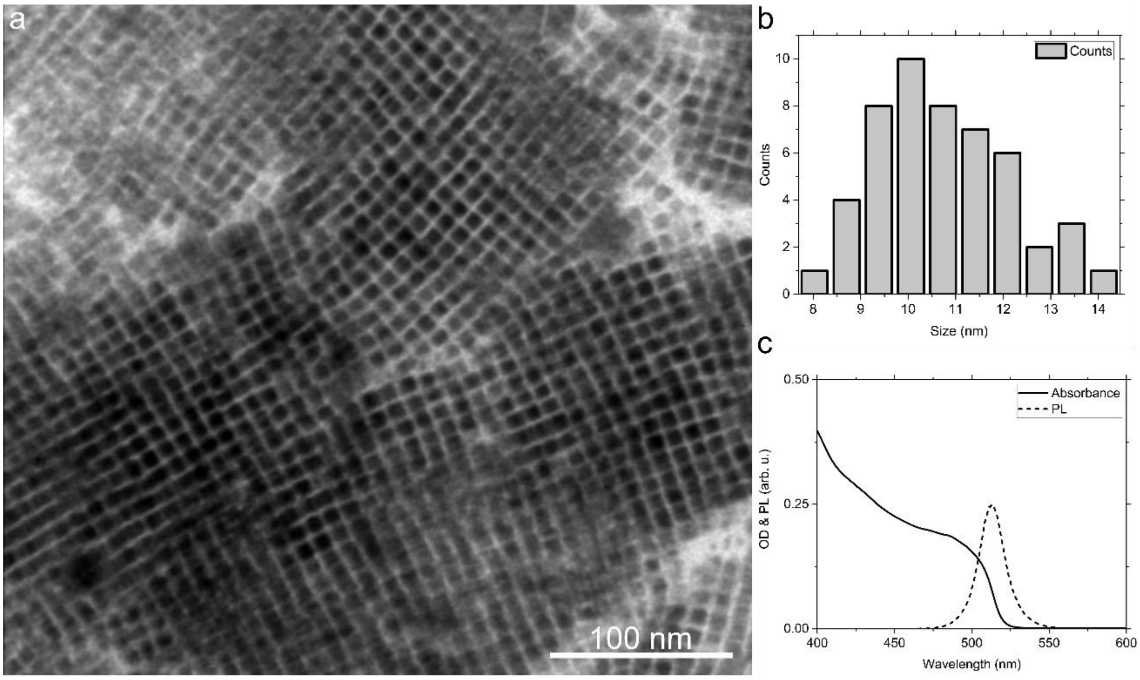

Figure S1. Size and emission of CsPbBr3 nanocrystals (a) TEM image of the NC dispersion used for the fabrication of photonic crystals. The NCs assemble already on the TEM grid and form ordered superlattice arrangements. b) Size distribution of NCs obtained from a TEM image gives an average size of 10.7 nm with a standard deviation of 0.9 nm. c) The nanocrystal dispersion shows photoluminescence at a center wavelength of 513 nm (2.415 eV) with a Gaussian FWHM of 21 nm (98 meV). The optical density of the NC dispersion reveals a small enough exciton binding energy so that exciton states and continuum onset overlay.

5. PDMS Molds

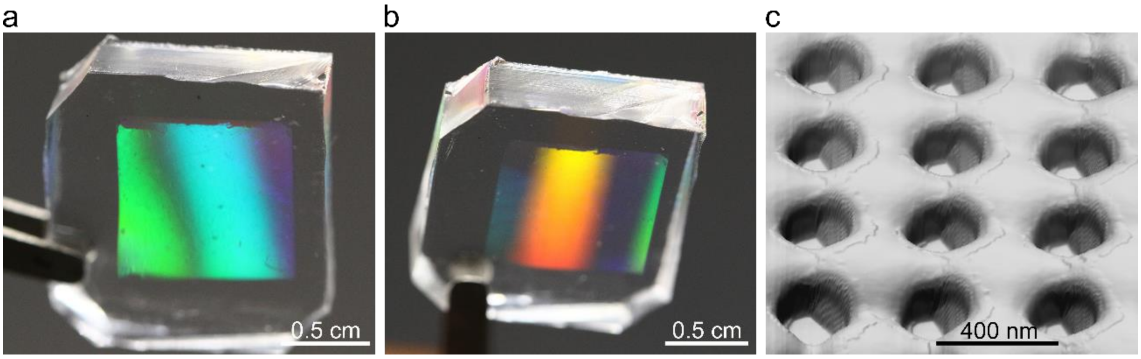

Figure S2. Reusability of PDMS Molds (a,b) Macroscopic images of the PDMS molds showing iridescence form the patterned area (1 cm^2) under different angles and (c) AFM 3D image of the array of holes of a PDMS mold after several uses as template for the NCs self-assembly. The AFM characterization of the PDMS pattern, together with SEM imaging of the produced photonic crystals, confirm the reusability of the mold.

6. Low magnification images of Perovskite Photonic Crystals

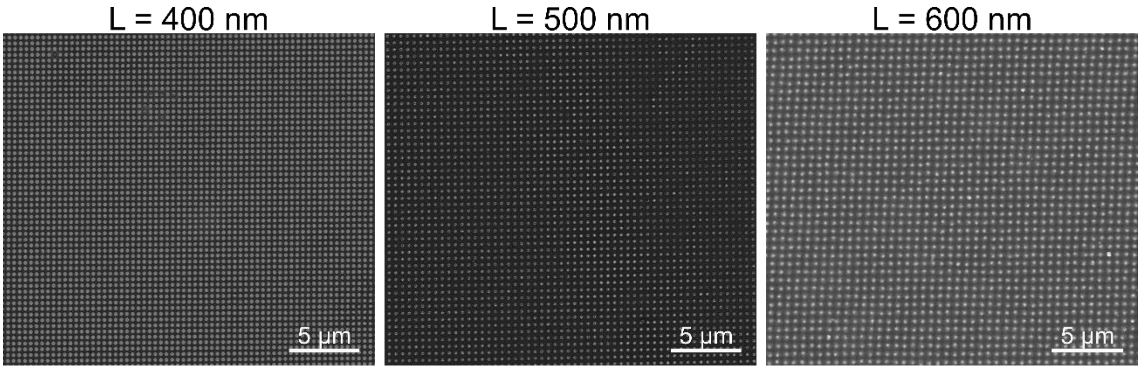

Figure S3. Library of lattice parameters. Low-magnification SEM images of photonic crystals of lattice parameters of 400, 500 and 600 nm. In all cases, the self-assembly technique proves to be suitable for building homogeneous nanostructures in a periodical array with almost no defects.

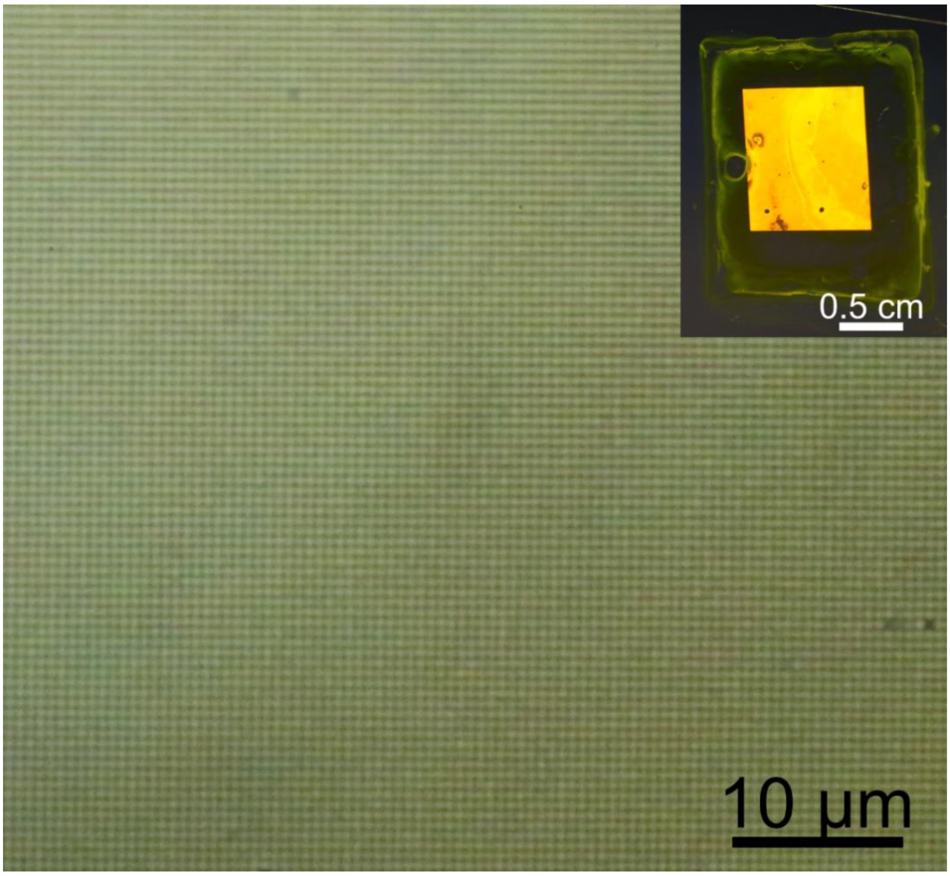

Figure S4. Large-area homogeneity Bright field optical microscopy image (100x) of a sample of 600 nm of lattice parameter. The pillars can be resolved showing a uniform and large-area 2D photonic crystal. Inset: macroscopic image of the photonic crystal on a glass substrate.

7. TEM and SEM inspection of Perovskite Photonic Crystals

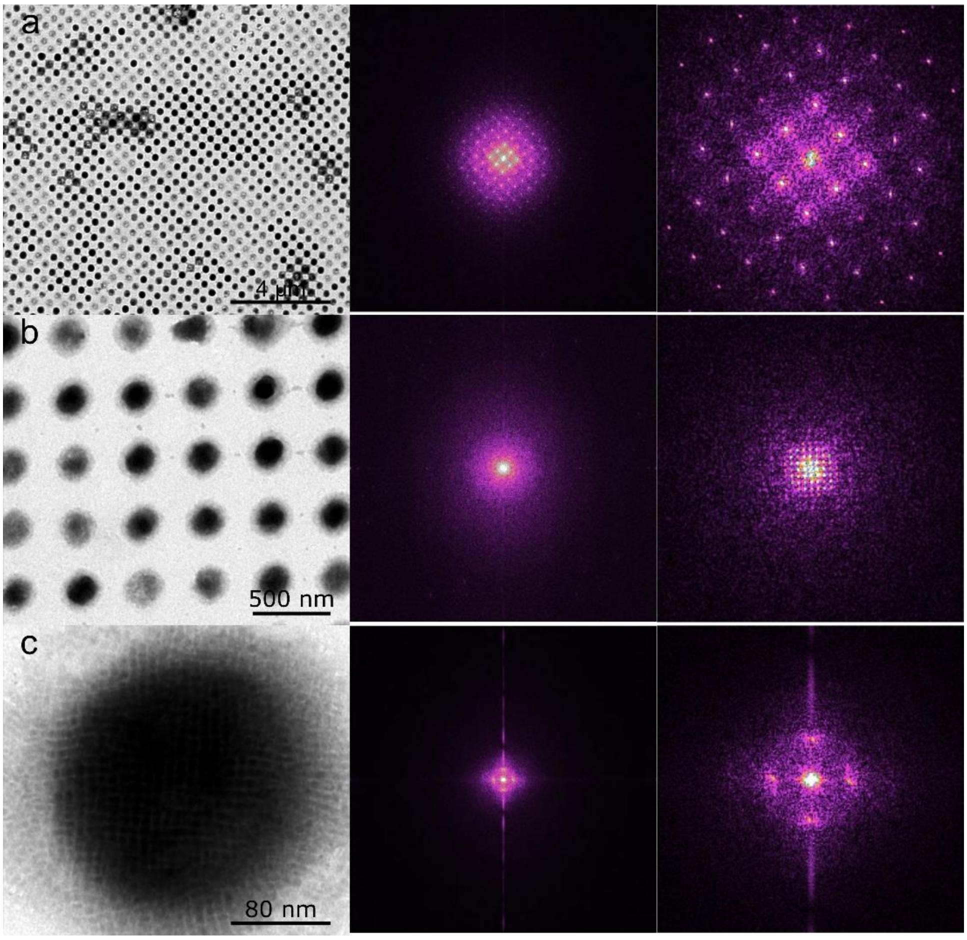

Figure S5. Assembly of NCs on TEM grids. TEM images at different magnifications of an array of structures made by applying the optimized self-assembly technique using a TEM grid as substrate. High magnification imaging of a pillar consisting of just a few layers (c) allows to distinguish the individual NCs, which appear to be arranged in a cubic close-packed structure. 2D Fast Fourier Transform (FFT) analysis of the TEM images and a 4x zoom of it (mid and right columns, respectively) confirm the periodicity of both, the clusters building a photonic crystal and the ordered nanocrystals inside each cluster.

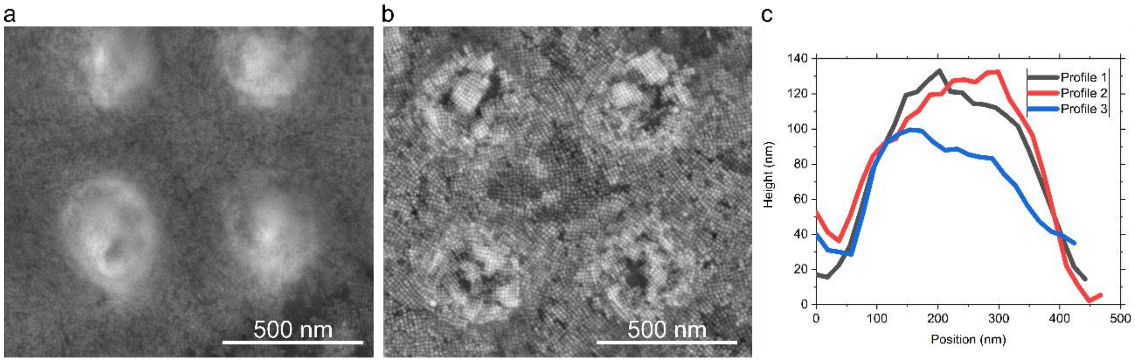

Figure S6. Effect of presence of free ligands. SEM images showing photonic crystal prepared from (a) NCs dispersion that contained excess of free ligands and (b) NCs dispersion after ligand purification. In the presence of free ligands (a), only the NCs in the residual layers between the pillars can be resolved while the NCs assembled into the pillars are embedded into an organic matrix that cannot be imaged by SEM. Otherwise, the assembled NCs that build the pillars from a purified dispersion can be resolved (b), and are found to form ordered domains. (c) AFM profiles of the pillars height of perovskite photonic crystals.

Presence of excess ligands render the films more insulating and harder to image by SEM. Therefore, it is important to remove the excess ligands from $CsPbBr_3$ colloidal solution followed by concentration by centrifugation to obtain the photonic crystals shown in Figure 1 (see experimental section).

8. Photoluminescence of a flat NC film under 800 nm excitation.

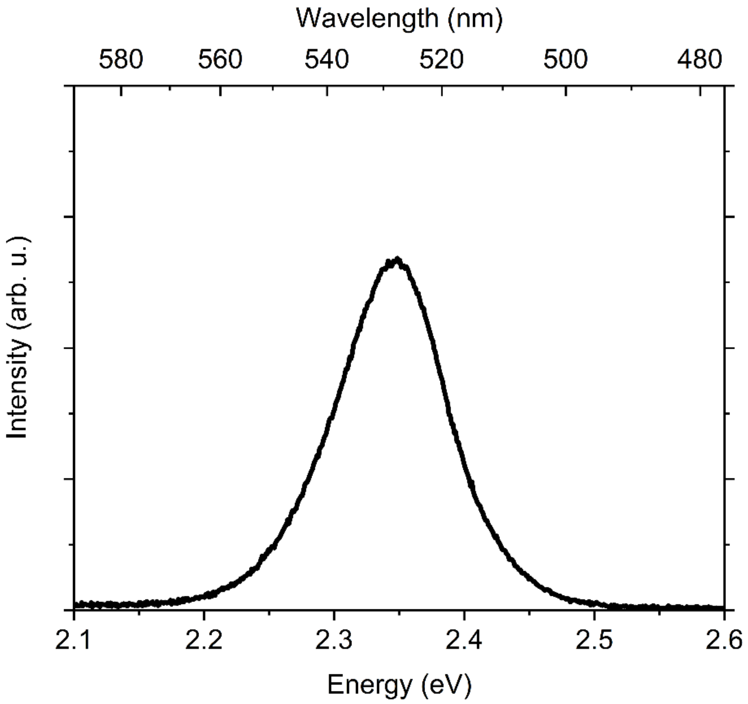

Figure S7. Photoluminescence of a flat NCs film under 800 nm excitation. Photoluminescence obtained from a flat NC film under 800 nm excitation and fluence of 13mJ/cm^{-2}. No ASE was observed.

9. References

[1] a) Y. Tong, E. Bladt, M. F. Aygüler, A. Manzi, K. Z. Milowska, V. A. Hintermayr, P. Docampo, S. Bals, A. S. Urban, L. Polavarapu, J. Feldmann, *Angew. Chem. Int. Ed.* **2016**, *55*, 13887-13892; b) Y. Tong, E.-P. Yao, A. Manzi, E. Bladt, K. Wang, M. Döblinger, S. Bals, P. Müller-Buschbaum, A. S. Urban, L. Polavarapu, J. Feldmann, *Adv. Mater.* **2018**, *30*, 1801117.

[2] T. W. Odom, J. C. Love, D. B. Wolfe, K. E. Paul, G. M. Whitesides, *Langmuir* **2002**, *18*, 5314-5320.

[3] A. Espinha, C. Dore, C. Matricardi, M. I. Alonso, A. R. Goñi, A. Mihi, *Nat. Photonics* **2018**, *12*, 343-348.

[4] C. Hanske, E. H. Hill, D. Vila-Liarte, G. González-Rubio, C. Matricardi, A. Mihi, L. M. Liz-Marzán, *ACS Appl. Mater. Interfaces* **2019**, *11*, 11763-11771.

[5] R. D. Deegan, O. Bakajin, T. F. Dupont, G. Huber, S. R. Nagel, T. A. Witten, *Nature* **1997**, *389*, 827-829.

[6] J. N. Lee, C. Park, G. M. Whitesides, *Anal. Chem.* **2003**, *75*, 6544-6554.

[7] S. Yakunin, L. Protesescu, F. Krieg, M. I. Bodnarchuk, G. Nedelcu, M. Humer, G. De Luca, M. Fiebig, W. Heiss, M. V. Kovalenko, *Nat. Commun.* **2015**, *6*, 8056.

[8] S. S. Wang, R. Magnusson, *Appl. Opt.* **1993**, *32*, 2606-2613.
